# The complete chloroplast genome of *Medicago scutellata* (Fabaceae)

**DOI:** 10.1080/23802359.2022.2039083

**Published:** 2022-02-16

**Authors:** Xiaofan He, Yingxue Jiao, Yuhua Shen, Tiejun Zhang

**Affiliations:** a School of Grassland Science, Beijing Forestry University, Beijing, China; b College of Chemistry and Life Sciences, Chifeng University, Chifeng, China

**Keywords:** Chloroplast genome, *Medicago scutellata*, Fabaceae

## Abstract

*Medicago scutellata* (Linnaeus, 1753) is one of the essential leguminous forages distributed across tropical and subtropical regions of the world. In our study, we obtained the complete chloroplast genome of *M*. *scutellata* with a length of 124,082 bp. The total GC content of the entire chloroplast genome of *M*. *scutellata* was 33.9%. Among the 110 unique genes in the circular genome, 30 tRNA, 4 rRNA, and 76 protein-coding genes were successfully annotated. A phylogenetic tree constructed using common protein-coding genes revealed that *M*. *scutellata is* closely related to *M. truncatula* from the Fabaceae family.

*Medicago scutellata* (Linnaeus, 1753) is a major annual legume that is widely cultivated throughout the world, particularly in the Mediterranean region. It is a highly productive forage crop that is also stress tolerant, making it an excellent source and valuable forage crop for livestock fodder (Khaef and Sadeghi 2011). In addition, the seeds of *M*. *scutellata* also contain significantly higher trypsin inhibitor levels than other *Medicago* species, suggesting that *M. scutellata* could act as a potentiator of cisplatin-mediated trypsin inhibitor as a plant-derived anticancer agent for human breast and cervical cancers (Lanza et al. 2004). It also possesses extensive glandular stems and leaf hairs that serve as an anthelmintic mechanism and provide resistance against insects (Bauchan 1987).

Chloroplast is a vital organelle that has its own DNA genome and plays an important role in the fixation of carbon dioxide and the regulation of several metabolic pathways. The chloroplast genome of plants has been a focus of research in plant molecular evolutionary and phylogenetic studies (Clegg et al. 1994). In addition, the availability of the complete chloroplast genome sequences can facilitate the development of chloroplast transformation technology (Bock 2001). Therefore, the chloroplast genome needs to be investigated and analyzed in greater depth. However, so far, the chloroplast genome sequence of *M*. *scutellata* has not been reported. In the present study, the chloroplast genes of *M*. *scutellata* were sequenced and structurally characterized, providing a valuable resource for further studies on the genetic evolution of legumes, especially fodder crops. The chloroplast genome sequencing of *M. scutellata* will serve as a solid basis for molecular biology, genetic breeding of *M*. *scutellate*, and the development of new fodder crops (Tao et al. 2017).

The sample of *M*. *scutellata* was collected from the Bajia Botanical Garden (E116°29′, N40°03′) in Beijing, China. Seeds of *M*. *scutellata* were kept in the Forage Germplasm Bank of the School of Grassland Science, Beijing Forestry University (BFU) for conservation and available for use in research. And the voucher specimen were deposited in the Herbarium of Beijing Forestry University (http://cxy.bjfu.edu.cn/, Tiejun Zhang, tiejunzhang@126.com) under the voucher number MS535644. In the laboratory, leaves were collected from germinated plants, and genomic DNA was extracted using a DNA extraction kit from Shanghai Limin Industrial Co., Ltd (Shanghai, China). Sequencing was performed on the Illumina Novaseq PE150 platform (Illumina Inc, San Diego, USA) to generate 150 bp paired-end reads. The cleaned reads were assembled into complete chloroplast genomes using the software GetOrganelle v1.5 (Jin et al. 2018), using the previously described chloroplast genome of *Medicago truncatula* (GenBank accession number: NC_003119) as a reference. Annotation of the chloroplast genome was performed using the online programs CPGAVAS2 (Shi et al. 2019) and GeSeq (Tillich et al. 2017), followed by manual correction. The annotated chloroplast genome sequences were registered in GenBank under the accession number MZ895077. The study on *M*. *scutellata*, including the collection of plant material was carried out in accordance with guidelines provided by School of Grassland Science, Beijing Forestry University and Chinese or international regulations. Field studies has followed all the research protocols and complied with the legislation of Beijing.

In this study, the complete chloroplast genome of *M*. *scutellata* was determined to be 124,082 bp in length. The GC content of the entire chloroplast genome was 33.9%. A total of 110 genes were identified in the chloroplast genome, including 76 protein-coding genes, 30 tRNA genes, and 4 rRNA genes. Among them, 30 genes encoding amino acid transfer proteins, 15 genes encoding light-harvesting structural proteins of PSII, 11 genes encoding *NADH* dehydrogenase proteins, 11 genes encoding ribosomal proteins of the small subuni, were found in the chloroplast genome of *M*. *scutellata.*

The chloroplast genomes of 17 species from the Fabaceae family and *Melilotus albus* and *Trifolium repens* as outgroup species were downloaded from the National Center for Biotechnology Information (NCBI) GenBank database to determine the phylogenetic relationships of *M*. *scutellata.* These sequences were aligned using MAFFT v7 (Katoh et al. 2019). In addition, a maximum likelihood (ML) tree based on the common protein-coding genes of 19 species was constructed using raxmlGUI1.5b (v8.2.10) (Silvestro and Michalak 2012). According to the results of the phylogenetic study, the entire tree was divided into three main clades. Two outgroup specie, *Melilotus albus* and *Trifolium repens,* formed two independent branches. Seventeen species from the *Medicago* group belonged to one clade. And *M. truncatula* was shown to be closely related to *M. scutellata* (Figure 1). This study will provide valuable markers for species identification, phylogenetic relationships, and population genetics in the Fabaceae family, particularly for legume forage, as well as for other applications.

**Figure 1.**
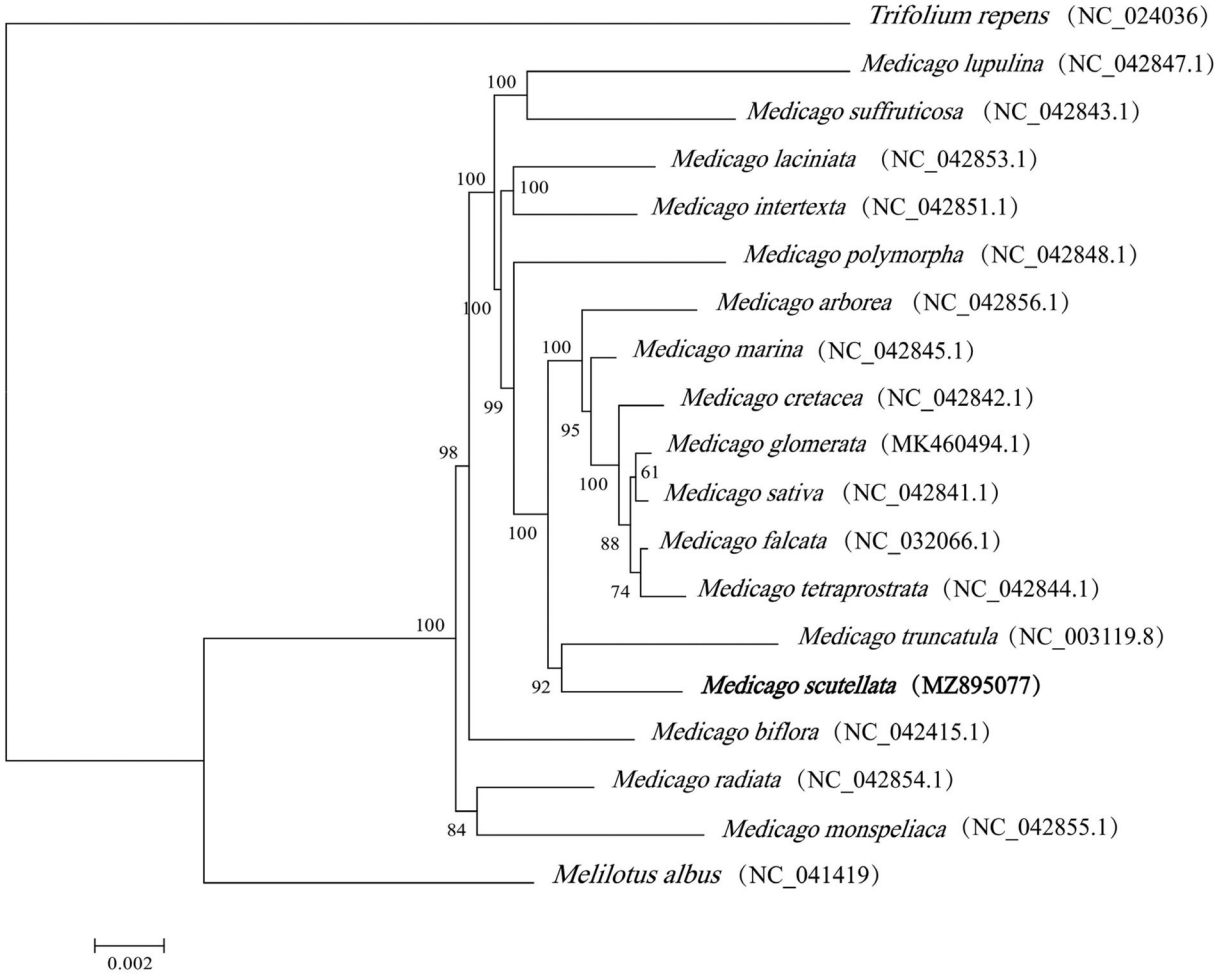
Phylogenetic tree reconstructed by the maximum likelihood (ML) method, based on common protein‐coding genes of 19 species of the Fabaceae family, with *Melilotus albus* and *Trifolium repens,* the sister group of *Medicago* within Fabaceae as outgroup. The numbers on the lines represent ML bootstrap values (>70%).

## Data Availability

The genome sequence data that support the findings of this study are openly available in GenBank of NCBI (https://www.ncbi.nlm.nih.gov/) under the accession no. MZ895077. The associated BioProject, SRA, and Bio-Sample numbers are PRJNA743404, SRR15390011, and SAMN19988356, respectively
